# Clinical and surveillance outcomes of the *TP53* c.1000G > C (p.Gly334Arg) variant

**DOI:** 10.1007/s10689-026-00591-y

**Published:** 2026-07-22

**Authors:** Yehudit Peerless, Rinat Bernstein-Molho, Iris Kventsel, Zehavit Frenkel, Lilach Levi, Inbal Kedar, Aviad Zick, Tamar Peretz, Yael Goldberg, Naama Halpern

**Affiliations:** 1 The Jusidman Cancer Center, Sheba Medical Center, Ramat Gan, Israel; 2 The Suzanne Levy-Gertner Oncogenetics Unit, Danek Gertner Institute of Human Genetics, Sheba Medical Center, Ramat Gan, Israel; 3 Gray Faculty of Medical and Health Sciences, School of Medicine, Tel-Aviv University, Tel-Aviv, Israel; 4 Department of Pediatric Hemato-Oncology, Edmond and Lilly Safra Children’s Hospital and Cancer Research Center, Sheba Medical Center, Ramat Gan, Israel; 5 Rabin Medical Center, Recanati Genetics Institute, Beilinson Hospital, Petah Tikva, Israel; 6 Department of Oncology, Sharett Institute of Oncology, Hadassah Medical Center, Jerusalem, Israel; 7 Faculty of Medicine, Hebrew University, Jerusalem, Israel

**Keywords:** *TP53*, Li-Fraumeni syndrome, Ashkenazi Jewish, Whole-body MRI, Cancer surveillance

## Abstract

**Background:**

Germline *TP53* pathogenic variants are classically associated with Li-Fraumeni syndrome, although penetrance and tumor spectrum vary substantially across specific alleles. The Ashkenazi Jewish *TP53* c.1000G > C (p.Gly334Arg) variant has been reported as a lower-penetrance, later-onset cancer predisposition allele, but its clinical classification and penetrance remain subjects of ongoing discussion. Consequently, the optimal surveillance approach for carriers remains uncertain, and real-world surveillance outcome data are limited.

**Methods:**

We performed a retrospective descriptive study of heterozygous *TP53* p.Gly334Arg carriers followed at two Israeli hereditary cancer clinics between 2021 and 2025. Clinical records were reviewed for demographic data, personal and family cancer history, and surveillance outcomes. Whole-body magnetic resonance imaging (WB-MRI) findings were analyzed for carriers who underwent serial surveillance.

**Results:**

Eighteen carriers from five families were identified, all of Ashkenazi Jewish ancestry. Seven of 18 carriers (39%) had a personal history of malignancy. Breast cancer was the most common malignancy, occurring in four women, all diagnosed after age 50; available pathology showed hormone receptor-positive disease. No pediatric malignancies were observed, and none of the carriers met classic or Chompret clinical criteria for Li-Fraumeni syndrome. Ten carriers underwent serial WB-MRI surveillance, totaling 36 examinations (mean 3.6 per patient). No malignancies were detected. However, surveillance generated nine abnormal findings (0.25 per scan), all false positives, including one that led to invasive procedures.

**Conclusions:**

In this clinic-based cohort, *TP53* p.Gly334Arg was observed in association with a later-onset, potentially lower-penetrance cancer phenotype and low short-term surveillance yield. These findings support phenotypic heterogeneity among *TP53* variants and raise questions regarding the optimal intensity of surveillance in this population; however, current data are insufficient to support modification of existing *TP53* surveillance protocols. Larger multicenter studies with longer follow-up are needed to define penetrance and guide genotype-informed management.

## Introduction

Germline pathogenic variants in *TP53* [OMIM #151623] underlie Li-Fraumeni syndrome (LFS), a highly penetrant hereditary cancer predisposition syndrome characterized by a broad spectrum of pediatric- and adult-onset malignancies, including sarcomas, breast cancer, brain tumors, leukemia, and adrenocortical carcinoma (ACC) [1–3]. With expanding germline testing, it has become increasingly clear that not all pathogenic *TP53* variants confer equivalent risk. The broader concept of heritable *TP53*-related cancer syndrome reflects this clinical and biological heterogeneity [4–6].

Surveillance has become a central component of management for *TP53* variant carriers. Prospective studies have shown that structured surveillance, including whole-body magnetic resonance imaging (WB-MRI), can facilitate early tumor detection and improve outcomes in appropriately selected carriers [7]. Current expert guidelines therefore recommend intensive surveillance for most individuals with pathogenic germline *TP53* variants [4, 8]. However, whether all *TP53* variants warrant uniform surveillance intensity remains an unresolved and clinically consequential question, particularly for alleles associated with attenuated penetrance [5, 6]. The Ashkenazi Jewish *TP53* c.1000G > C (p.Gly334Arg) variant has emerged as one such allele. Powers et al. described it as a pathogenic low-penetrance variant associated with multiple cancers, typically at older ages than seen in classic LFS [9]. However, interpretation of *TP53* p.Gly334Arg remains a matter of ongoing discussion. Although several groups have classified the variant as pathogenic or likely pathogenic, other expert classifications have designated it as a variant of uncertain significance [9–11]. 

Consequently, the present study evaluates the clinical phenotype and surveillance outcomes observed among carriers rather than attempting to resolve variant classification. This raises important questions regarding phenotype definition, ascertainment, and the balance between surveillance benefit and burden.

Founder and ancestry-enriched *TP53* variants in other populations further illustrate this heterogeneity. The Brazilian c.1010G > A (p.Arg337His) variant is associated with a broad but variably penetrant tumor spectrum, including childhood ACC [12, 13]. The Arab-Muslim c.541C > T (p.Arg181Cys) variant appears attenuated relative to classic LFS but still includes pediatric and core *TP53*-associated malignancies [14]. More recently, the c.542G > A (p.Arg181His) variant, enriched in a Swedish cohort**,** has been associated with a more restricted breast- and prostate-predominant adult phenotype, prompting discussion of phenotype-adapted surveillance [15]. In this context, real-world surveillance outcomes in *TP53* p.Gly334Arg carriers are important to inform whether uniform application of intensive screening protocols remains appropriate across heterogeneous* TP53* genotypes.

Here, we report the clinical and surveillance characteristics of 18 additional carriers of the Ashkenazi Jewish *TP53* p.Gly334Arg variant followed at two Israeli hereditary cancer clinics, with particular emphasis on malignancy patterns and longitudinal WB-MRI outcomes.

## Methods

### Study design and setting

This was a retrospective descriptive cohort study of heterozygous carriers of *TP53* p.Gly334Arg variant evaluated at two hereditary cancer clinics in Israel between January 2021 and January 2025.

### Participants

Eligible individuals were heterozygous carriers of *TP53* c.1000G > C (p.Gly334Arg) identified through clinical multigene panel testing or cascade testing for a known familial *TP53* p.Gly334Arg variant and followed within one of the participating hereditary cancer clinics. The participating institutions serve as referral centers for hereditary cancer surveillance and therefore follow both locally identified carriers and individuals referred after genetic testing performed elsewhere. During the study period, approximately 150 individuals carrying germline *TP53* variants were under surveillance across the two centers. One of the participating centers includes a dedicated pediatric surveillance program for *TP53* carriers, while both centers maintain detailed family cancer history documentation as part of routine clinical assessment. All available *TP53* p.Gly334Arg carriers were included, resulting in a cohort of 18 carriers from five families. Probands underwent comprehensive germline testing, whereas relatives were generally identified through familial cascade testing for the known *TP53* p.Gly334Arg variant. Additional hereditary cancer predisposition gene testing was performed in some individuals as part of routine clinical care, although such testing was not uniformly available across the entire cohort.

### Data collection

Families were evaluated for fulfillment of classic Li-Fraumeni syndrome and modified Chompret criteria according to established published definitions [5]. Electronic medical records were reviewed for demographic characteristics, ancestry, personal history of malignancy, age at cancer diagnosis, available tumor pathology, family history, and clinical criteria relevant to LFS assessment. Surveillance data were collected for carriers who underwent serial WB-MRI examinations, including number of scans, interval follow-up, abnormal findings, and downstream investigations.

### Outcomes

The primary descriptive outcomes were distribution of malignancies, age at diagnosis, and WB-MRI surveillance yield. Secondary outcomes included the frequency of incidental or indeterminate findings requiring additional work-up.

### Statistical analysis

Given the small cohort size and descriptive purpose of the study, no formal hypothesis testing was planned. Data are presented as counts, percentages, ranges, and means where appropriate.

### Ethics

The study was conducted in accordance with institutional requirements and approved by the relevant ethics committees of the participating institutions. Patient data were reviewed retrospectively in de-identified form in accordance with local regulations.

## Results

### Cohort characteristics

Eighteen *TP53* p.Gly334Arg carriers from five families were identified (Table 1). All were of Ashkenazi Jewish ancestry. The mean age at last follow-up was 52.6 (range 22–78), and the cohort included 7 males and 11 females. None met classic LFS or Chompret clinical criteria based on the available clinical data.

**Table 1:** Clinical characteristics of 18 carriers of *TP53* c.1000G > C (p.Gly334Arg)

Family ID	Age, y	Sex	Personal cancer history	Breast cancer subtype (if applicable)	Family history (unknown carrier status)	Chompret criteria	Classic LFS criteria	Cancer-free carrier at last follow-up
1a	44	M	No	NA	Breast cancer (age unknown)	No	No	Yes
1b	78	F	Breast cancer at 53	HR +	NA	—	—	No
1c	52	F	No	NA	NA	—	—	Yes
1d	NA	F	No	NA	NA	—	—	Yes
2a	72	F	Breast cancer at 57	HR +	Breast cancer (age 50, × 2)	No	No	No
2b	38	F	No	NA	NA	—	—	Yes
2c	NA	M	No	NA	NA	—	—	Yes
3a	59	F	Neuroendocrine Tumor of Small bowel at 54	NA	NA	No	No	No
3b	NA	M	Prostate and kidney cancers (> 60)	NA	NA	—	—	No
4a	59	F	Breast cancer at 53	HR +	Prostate cancer (age 70)	No	No	No
4b	22	F	No	NA	NA	—	—	Yes
4c	65	M	No	NA	NA	—	—	Yes
4d	31	F	No	NA	NA	—	—	Yes
4e	64	F	Breast cancer at 57	HR +	NA	—	—	No
4f	33	M	No	NA	NA	—	—	Yes
4g	NA	F	No	NA	NA	—	—	Yes
5a	76	M	Papillary thyroid carcinoma at 56; sarcoma at 66	NA	Uterine cancer (age 85); lung cancer (ages 56–70); pancreatic cancer (age 83)	No	No	No
5b	43	M	No	NA	NA	—	—	Yes

HR + , hormone receptor positive, NA indicates not available, — indicates same family-level assessment as the first listed member of that family, Chompret and classic Li-Fraumeni syndrome criteria are family-level assessments and are reported once per family

### Personal history of cancer

Seven of 18 carriers (39%) had a personal history of malignancy (Table 1). All reported cancers occurred in adulthood, and all first malignancies occurred after age 50 in this cohort.

Two carriers had multiple primary malignancies.

Breast cancer was the predominant tumor type in female carriers and represents an important clinical feature of this cohort. All four affected women were diagnosed after age 50, specifically between ages 53 and 57. Pathological data were available for all four breast cancer cases, and all tumors were hormone receptor-positive and HER2-negative.

No pediatric malignancies were observed in this cohort.

### Surveillance outcomes

Ten carriers underwent serial WB-MRI surveillance. Across these individuals, 36 WB-MRI examinations were performed, with a mean of 3.6 scans per patient. No malignancies were detected during the observed follow-up period.

Surveillance generated a measurable burden of incidental findings. Nine abnormal findings were identified across 36 examinations, all of which proved benign after additional evaluation. One finding prompted invasive investigation, including lumbar puncture and biopsy, while the remainder required noninvasive follow-up imaging. Eight carriers either did not undergo WB-MRI surveillance, underwent surveillance at external centers where data were unavailable, or declined participation, resulting in heterogeneous surveillance exposure across the cohort.

## Discussion

This clinic-based series expands the available clinical data on the Ashkenazi Jewish *TP53* p.Gly334Arg variant. None of the carriers in our cohort met classic or Chompret clinical criteria, and all cancers occurred in adulthood. To our knowledge, this study represents the first independent clinical series reported since the initial characterization of *TP53* p.Gly334Arg by Powers et al. [9]. Powers et al. identified 22 probands carrying the *TP53* p.Gly334Arg variant from multiple predominantly Ashkenazi Jewish families and additionally reported clinical data from affected relatives, including mutation carriers. Most affected individuals developed adult-onset malignancies, including breast cancer, hematologic malignancies, sarcoma, brain tumors, and other LFS-spectrum cancers, while six individuals from four families developed pediatric ACC. Eight individuals from four families had multiple primary tumors. Importantly, the median age at onset of both overall cancer and breast cancer was significantly later than that observed in classic Li-Fraumeni syndrome cohorts, despite a similar lifetime cumulative cancer incidence. The authors therefore proposed a variable phenotype characterized by later-onset cancers in many families while retaining the potential for childhood malignancies in a subset of carriers [9]. Our findings are broadly consistent with these observations, with breast cancer representing the most common malignancy among women and two carriers developing multiple primary cancers. However, in contrast to the Powers cohort, no pediatric malignancies, including ACC, were observed in our series, although the limited sample size precludes definitive conclusions regarding childhood cancer risk.

Among female carriers in our cohort, breast cancer was the most common malignancy observed. Notably, all breast cancers occurred after age 50, and available pathology showed hormone receptor-positive/HER2-negative tumors. This pattern differs from the classic LFS breast cancer phenotype, which is typically earlier in onset and enriched for HER2-positive disease [16]. Although pathology review was not centralized, and detailed tumor data were incomplete for some cases, the available findings suggest that breast cancers arising in *TP53* p.Gly334Arg carriers may differ from the breast cancer phenotype traditionally associated with classic Li-Fraumeni syndrome.

The absence of pediatric malignancies in this cohort should be interpreted cautiously. Although one participating center includes a dedicated pediatric *TP53* surveillance program and family histories were reviewed across both centers, definitive conclusions regarding childhood cancer risk cannot be drawn. The discrepancy between our findings and those reported by Powers et al. [9] may reflect limited sample size, referral patterns, incomplete family ascertainment, or chance. The surveillance findings in this cohort also warrant consideration. No malignancies were detected during follow-up in our cohort, although serial WB-MRI examinations generated a measurable burden of benign follow-up investigations. These observations extend the limited available data regarding surveillance outcomes in *TP53* p.Gly334Arg carriers. The limited number of participants and duration of follow-up preclude conclusions regarding surveillance yield, and current evidence remains insufficient to support modification of existing *TP53* surveillance recommendations.

Several limitations should be acknowledged. This was a small retrospective study from two specialized clinics and is therefore vulnerable to referral and ascertainment bias. Follow-up duration was limited, surveillance participation was not uniform across all carriers, and pathology details were incomplete for some tumors. Family-based segregation data and centralized tumor review were not available. In addition, classification of *TP53* p.Gly334Arg remains a subject of ongoing discussion, with some expert classifications designating the variant as of uncertain significance while others consider it likely pathogenic or pathogenic. Accordingly, the present findings should be interpreted as observations among carriers of this allele rather than as evidence regarding variant classification itself. These limitations restrict inference regarding penetrance and preclude recommendations for modified surveillance.

Despite these limitations, this study provides additional independent clinical and surveillance data for *TP53* p.Gly334Arg carriers, extending the limited evidence available for this rare Ashkenazi founder variant.

## Conclusion

The present findings are consistent with previous evidence suggesting that *TP53* p.Gly334Arg is associated with a later-onset, potentially lower-penetrance cancer susceptibility phenotype compared with classic Li-Fraumeni syndrome. In this clinic-based cohort, all malignancies occurred in adulthood, and no pediatric malignancies were observed, although this finding should be interpreted cautiously. Larger collaborative studies with longer follow-up are needed to better define variant-specific cancer risks and determine whether genotype-informed risk stratification may ultimately refine clinical management.

## Data Availability

The datasets generated during and/or analyzed during the current study are available from the corresponding author upon reasonable request.

## Abbreviations

ACC: Adrenocortical carcinoma
CNS: Central nervous system
LFS: Li-Fraumeni syndrome
WB-MRI: Whole-body magnetic resonance imaging
